# Correlation analysis of the platelet-to-lymphocyte ratio with attention-deficit hyperactivity disorder in children aged 6–14: based on the National Health and Nutrition Examination Survey database

**DOI:** 10.1017/S1092852924002475

**Published:** 2025-01-06

**Authors:** Xianhua Li, Yan Luo, Xiaodan Zeng, Xuewen Qiu, Yang Hua, Lingling Zhan

**Affiliations:** Paediatrician, Longyan First Hospital Affiliated to Fujian Medical University, Longyan, Fujian, China

**Keywords:** platelet-to-lymphocyte ratio, attention deficit hyperactivity disorder, inflammation, National Health and Nutrition Examination Survey

## Abstract

**Objective:**

This study aimed to elucidate the association between the platelet-to-lymphocyte ratio (PLR) and the risk of attention deficit hyperactivity disorder (ADHD) in children aged 6–14 based on National Health and Nutrition Examination Survey (NHANES) data.

**Method:**

We utilized data from NHANES 2013–2014 for analysis, with PLR as the independent variable and ADHD as the dependent variable. Weighted logistic regression was used to construct the relationship model. The subgroup analysis with stratification and adjustment for confounding factors was conducted to explore the association between PLR and ADHD risk in children aged 6–14. Finally, the restricted cubic spline (RCS) analysis was carried out to explore the non-linear relationship between PLR and ADHD.

**Results:**

The study included 1455 samples with 91 ADHD cases. A significant positive association (OR > 1, *P* < 0.05) was observed between PLR and ADHD risk in the multivariate weighted logistic regression model. Race and asthma status remarkably influenced the relationship between PLR and ADHD (*P* for interaction<0.05). The positive association between PLR and ADHD risk was particularly significant (*P* < 0.05) in boys, children born to mothers aged 20–29, and children with asthma. The RCS curve indicated a non-linear association between PLR and ADHD risk (*P*-non-linear = 0.0040), with OR > 1 when PLR ≥ 106.40.

**Conclusion:**

Increased PLR elevated the risk of ADHD, especially in males, children born to mothers aged 20–29, and children with asthma, with 106.40 possibly being an effective threshold for PLR’s impact on ADHD risk.

## 1. Introduction

Attention deficit hyperactivity disorder (ADHD) is the most common neurodevelopmental disorder diagnosed in childhood, characterized by symptoms such as inattention, hyperactivity, and impulsivity, which deviate from typical developmental patterns.^1^ The global prevalence of ADHD in children ranges from 2% to 7%.^2^ Although it has long been considered a childhood disorder, in most cases it persists into adulthood.^3^ Children with ADHD face many challenges, including poor cognitive and learning abilities,^4^ as well as higher rates of mental disorders and more healthcare service requirements.^5^ ^,^ ^6^ ADHD is a costly disease for society, with an estimated 6.1–16.3% of children and adolescents in the United States affected by ADHD.^7^ The annual social excess cost associated with ADHD is as high as $33.2 billion.^8^ Therefore, identifying potential factors related to ADHD is of significant importance for preventing and reducing the burden of the disease.

However, merely identifying and intervening based on symptoms is insufficient to effectively reduce the incidence and economic costs of the disease. In recent years, research has begun to focus on the potential role of inflammation in the pathogenesis of ADHD. Inflammation is a biological response characterized by elevated levels of acute-phase proteins and cytokines in circulation and increased infiltration of immune cells.^9^ Inflammation is tightly linked with developmental changes in the brain, and cytokines produced during inflammatory events can enter the brain barrier through various pathways and affect the central nervous system structure.^10^ There is increasing evidence proving that the inflammatory process is one of the main pathophysiological mechanisms of ADHD.^11^ Therefore, exploring the association between inflammatory markers and ADHD is particularly important.

The platelet/lymphocyte ratio (PLR) has attracted attention as a low-cost, simple, and applicable test for predicting clinical outcomes of chronic low-grade inflammation and neuroimmune diseases.^12^ ^–^^14^ Previous studies have demonstrated that PLR plays an essential role in the pathophysiology of ADHD in children, and it may be a potential inflammatory marker for childhood ADHD.^15^ ^,^ ^16^ These studies have mostly focused on ADHD children and adolescents aged 6–17,^17^ lacking specific research on children aged 6–14. However, the age range of 6–14 years is a crucial period for children’s neurodevelopment.^18^ ^,^ ^19^ Relevant studies indicate that ADHD is most common among children aged 6–12 years,^20^ with an increasing prevalence in the 10–14 age group.^2^ This suggests that children in this age range may face a higher risk of ADHD, and the inflammatory processes occurring during this developmental stage may play a crucial role in the onset and progression of the disease. Additionally, about 1/3 of mental disorders begin before the age of 14^21^; identifying and understanding inflammatory markers during this age range may also provide a key opportunity for early intervention and prevention. Therefore, focusing on this age group is crucial for developing targeted strategies for the prevention and treatment of ADHD.

Building on this, this study utilized the National Health and Nutrition Examination Survey (NHANES) database to investigate the association between PLR and ADHD in children aged 6–14. The aim was to uncover potential factors influencing this association and explore the potential role of PLR in the pathogenesis of ADHD. We hypothesized that an elevated PLR may be associated with an increased risk of developing ADHD.

## 2. Methods

### 2.1. Data source and study population

The NHANES database is a stratified, multi-stage study conducted by the National Center for Health Statistics (NCHS) of the United States (http://www.cdc.gov/nchs/nhanes.htm). This database is freely accessible and its main feature is to assess the health and nutritional status of the US population through a combination of interviews, physical examinations, and laboratory tests. The NHANES data obtained the approval of the NCHS Institutional Review Board and informed consent from participants.

The initial sample of this study included 10 175 respondents from the 2013–2014 cycle. To ensure the representativeness of the sample and the integrity of the data, we established the following inclusion and exclusion criteria:

Inclusion Criteria: (1) Children aged 6–14 years; (2) Complete data on platelet count and lymphocyte count; (3) Detailed ADHD history information and data on the use of ADHD-related prescription medications provided in the NHANES survey.

**Figure 1.**
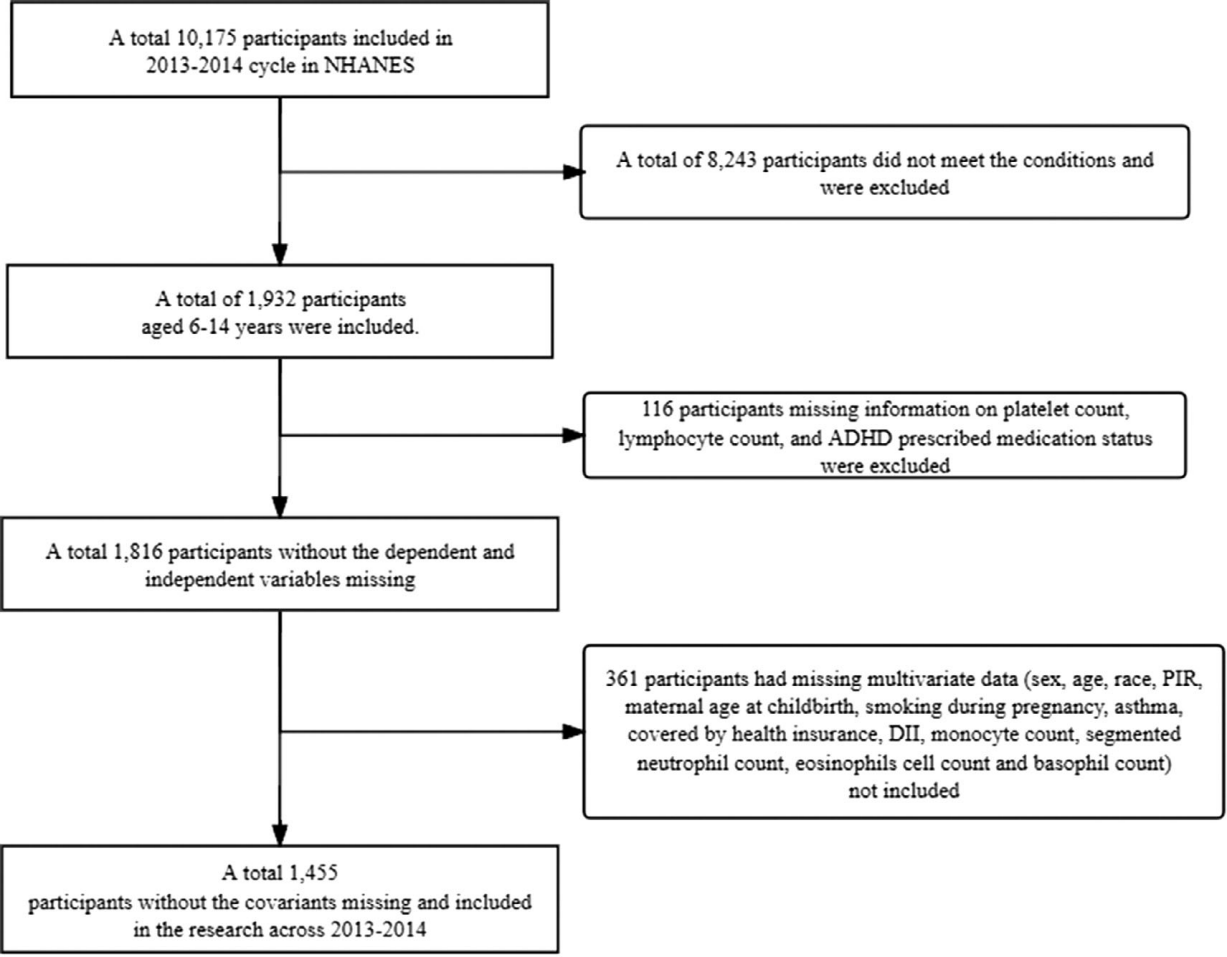
The flowchart of sample selection in NHANES 2013–2014.

Exclusion Criteria: (1) Respondents whose age did not meet the requirements, with a total of 8243 excluded; (2) Respondents with missing data on platelet count, lymphocyte count, or ADHD prescription medication status, with a total of 116 excluded; (3) Respondents with missing covariate data, with a total of 361 excluded. After this screening process, a total of 1455 eligible respondents were included in the study sample. The detailed screening process for this study is illustrated in Figure 1.

### 2.2. PLR

The PLR data collection was based on the Beckman Coulter DxH800 instrument in the NHANES Mobile Examination Center (MEC) for complete blood cell counts of all participants. The whole blood count analysis of platelet count and lymphocyte count was performed using the five-part classification. PLR was calculated using the formula of platelet count and lymphocyte count. PLR = platelet count/lymphocyte count.^22^

### 2.3. ADHD

The ADHD data collection was based on detailed information about medical history and prescription drugs obtained from participants in the NHANES questionnaire. Participants were clustered into two groups based on ICD-10-CM codes, one group being the ADHD patients with ADHD medication treatment, and the other group being the non-ADHD population (control group). The code of ICD-10-CM is F90 (F90: Attention-deficit hyperactivity disorders)^23^.

### 2.4. Variables

In this project, gender, race, poverty-to-income ratio (PIR), age, dietary inflammatory index (DII), maternal age at childbirth, smoking during pregnancy, health insurance status, asthma, monocyte count, eosinophil count, segmented neutrophil count, and basophil count were identified as potential confounding factors. The age of children selected for this experiment was limited to 6–14 years old. Race was classified as Mexican, other Hispanic, non-Hispanic white, non-Hispanic black, and other races. The classification criteria for PIR were ≤ 1.3 (low income), 1.3–3.5 (low to medium income), and > 3.5 (high income).^24^ The maternal age at childbirth and smoking status during pregnancy were based on personal interview data of children aged ≤15 years: (1) maternal age at childbirth: How old was your biological mother when you were born? (2) smoking status during pregnancy: Did your biological mother smoke during pregnancy? Asthma was defined as whether a doctor or other health professional had ever told you that you had asthma. If participants answered yes, they were considered asthma patients.^25^ Health insurance status was defined as whether the participant had health insurance or other types of healthcare plans, with answers “yes” or “no.” Blood samples from participants were obtained using the Beckman Coulter DxH 800 instrument in the MEC to obtain complete blood count (CBC) and blood cell distribution.^26^ The collected CBC information included monocyte count, segmented neutrophil count, eosinophil count, and basophil count.^27^

The DII data were based on two 24-hour dietary recalls conducted face-to-face in NHANES, followed by a telephone interview 3–10 days later to assess dietary intake. The 24-hour dietary recall questionnaire collected information on food types and portion sizes, while the food frequency questionnaire asked about food consumption frequency. The average intake of food was estimated as the average of two dietary recalls for each participant in the survey period. In this study, 28 food parameters were included to calculate the DII, including carbohydrates, proteins, alcohol, fiber, total fats, cholesterol, polyunsaturated fatty acids (PUFA), saturated fats, monounsaturated fatty acid (MUFA), n-3 fatty acids, niacin, vitamin A, n-6 fatty acids, thiamine (vitamin B1), riboflavin (vitamin B2), vitamin B12, vitamin B6, vitamin C, vitamin D, vitamin E, selenium, iron, magnesium, folate, beta-carotene, caffeine, zinc, and energy. Calculation steps: Z-score = (daily intake of a certain dietary component or nutrient - global mean daily intake) / standard deviation of the global mean daily intake of that dietary component or nutrient. To minimize the impact of right-skewness, the Z-score was converted to a percentile (minimizing the impact of outliers/right-skewness), and then the value was doubled and subtracted by 1 to achieve a symmetric distribution with 0 as the center. Finally, we multiplied the total inflammation score for each dietary component to calculate the inflammation index for each dietary component or nutrient to obtain the individual DII. Then, we added up the DII scores for the 28 foods to obtain the participants’ final DII scores. The DII scores ranged from negative to positive, with lower DII negative scores indicating anti-inflammatory effects and higher DII positive scores indicating pro-inflammatory effects.^28^ ^,^ ^29^ n-3 fatty acids included eicosatetraenoic acid (20:5), linolenic acid (18:3), clupanodonic acid (22:5), stearidonic acid (18:4), and docosahexaenoic acid (22:6). n-6 fatty acids included arachidonic acid (20:4) and linoleic acid (18:2).^30^

### 2.5. Statistical analysis

We employed the *Tableone* package to generate baseline tables. Based on the general population characteristics and whether they have ADHD, we grouped the respondents and drew baseline tables. Categorical variables were represented by n(%), while continuous variables were represented by mean(sd) (n: sample size without weight adjustment; n(%): proportion with weight adjustment; mean: mean value with weight adjustment; sd: standard deviation with weight adjustment). The *survey* package was utilized to construct a weighted logistic regression model of PLR and ADHD. The stratified analysis was carried out on the categorical variables in the model without adjusting for confounding factors. A likelihood ratio test was applied to the interaction term *P*-values of the stratified logistics regression model with all confounders adjusted. *P* < 0.05 indicates a significant difference. PLR was stratified by using weighted tertiles. The *survey* package was employed to construct a weighted logistic regression model of PLR and ADHD with adjusted confounding factors. We performed subgroup analysis on confounding factors and gender factors for interaction terms with *P* < 0.10 in the weighted logistic regression model of PLR and ADHD. In the weighted logistic regression model after adjusting for all confounding factors, restricted cubic splines (RCS) were utilized to investigate the association between PLR and ADHD. The models in this study included the Crude model without adjustment. Model I adjusted gender, age, race, PIR, maternal age at childbirth, smoking during pregnancy, asthma, and health insurance status. Model II adjusted gender, age, race, PIR, maternal age at childbirth, smoking during pregnancy, asthma, health insurance status, DII, monocyte count, segmented neutrophil count, eosinophil count, and basophil count. All statistical analyses were carried out by using R (V4.2.2) software.

## 3. Results

### 3.1. Baseline characteristics of the participants

A total of 1455 eligible participants were included in this project. The overall distribution of characteristics is shown in Table 1. There were 91 ADHD patients, with a mean age of 9.81 ± 2.34 years. The proportion of male patients was considerably higher (82.82% vs 54.10%, *P* = 0.004), and more patients had mothers who smoked during pregnancy (31.82% vs 13.28%, *P* = 0.002). The PLR value of children with ADHD (122.95 ± 36.64) was considerably higher than that of the non-ADHD population (106.86 ± 36.84, *P* = 0.002).

**Table 1.** Characteristics of NHANES participants between 2013–2014

Characters	Total	controls	ADHD medication users	*P* Value
Overall	1455	1364 (94.23)	91 (5.77)	
Gender				**0.004**
Female	670 (44.25)	658 (45.90)	12 (17.18)	
Male	785 (55.75)	706 (54.10)	79 (82.82)	
Age	10.10 (2.63)	10.11 (2.65)	9.81 (2.34)	0.306
Race				0.351
Mexican American	322 (15.07)	316 (15.53)	6 (7.66)	
Other Hispanic	145 (7.62)	137 (7.78)	8 (4.95)	
Non-Hispanic White	412 (53.99)	371 (53.47)	41 (62.43)	
Non-Hispanic Black	383 (14.81)	356 (14.58)	27 (18.51)	
Other race	193 (8.51)	184 (8.63)	9 (6.46)	
PIR				0.633
≤ 1.3	740 (39.03)	695 (38.75)	45 (43.64)	
1.3–3.5	459 (35.91)	424 (35.76)	35 (38.35)	
> 3.5	256 (25.06)	245 (25.49)	11 (18.01)	
Maternal age at childbirth				0.246
< 20	170 (9.58)	160 (9.45)	10 (11.60)	
20–29	840 (56.15)	778 (55.34)	62 (69.45)	
30–39	418 (32.12)	402 (32.96)	16 (18.45)	
≥ 40	27 (2.15)	24 (2.25)	3 (0.50)	
Smoking during pregnancy				**0.002**
No	1268 (85.65)	1201 (86.72)	67 (68.18)	
Yes	187 (14.35)	163 (13.28)	24 (31.82)	
Asthma				0.136
No	1082 (74.23)	1025 (75.04)	57 (60.93)	
Yes	373 (25.77)	339 (24.96)	34 (39.07)	
Covered by health insurance				0.147
No	95 (6.00)	94 (6.26)	1 (1.62)	
Yes	1360 (94.00)	1270 (93.74)	90 (98.38)	
DII	1.61 (1.59)	1.61 (1.59)	1.65 (1.65)	0.896
Monocyte (1000 cell/uL)	0.58 (0.19)	0.58 (0.19)	0.56 (0.18)	0.281
Neutrophil (1000 cell/uL)	3.66 (1.50)	3.68 (1.51)	3.32 (1.25)	0.073
Eosinophil (1000 cell/uL)	0.26 (0.26)	0.27 (0.27)	0.26 (0.19)	0.762
Basophil (1000 cell/uL)	0.04 (0.05)	0.04 (0.05)	0.04 (0.05)	0.932
PLR	107.79 (37.01)	106.86 (36.84)	122.95 (36.64)	**0.002**

DII, dietary inflammatory index; PLR, platelet-to-lymphocyte ratio.

*Note*: Categorical and continuous variables are expressed as n (%) and mean (sd), respectively. n: unweighted; n (%), mean and sd: weighted.

### 3.2. Stratified analysis

As shown in Table 2, stratified analysis of gender, race, maternal age at childbirth, smoking during pregnancy, PIR, asthma, and health insurance status indicated a positive correlation between PLR and the risk of ADHD in the population of boys, the population with PIR > 3.5, children born to mothers aged 20–39 at delivery, children born to non-smoking mothers during pregnancy, and children with asthma (OR > 1, *P* < 0.05). The interaction test between confounding factors and PLR suggested a significant interaction effect of race and asthma status on the correlation between PLR and ADHD (*P* for interaction<0.05).

**Table 2.** Relationship between PLR and ADHD medications in categorical variables

Participants	OR	95% CI1	*P*-value	*P* for interaction
Gender				0.129
Female	0.99	0.98–1.01	0.700	
Male	1.01	1.00–1.02	**0.006**	
Race				**0.023**
Mexican American	1.00	0.99–1.01	0.120	
Other Hispanic	0.99	0.97–1.02	0.400	
Non-Hispanic White	1.01	0.99–1.02	**0.041**	
Non-Hispanic Black	1.01	1.00–1.02	**0.002**	
Other race	1.03	1.01–1.04	**0.001**	
PIR				0.392
≤ 1.3	1.00	0.99–1.01	0.300	
1.3–3.5	1.01	0.99–1.02	**0.039**	
> 3.5	1.02	1.01–1.03	**<0.001**	
Maternal age at childbirth				0.053
< 20	1.00	0.99–1.01	0.300	
20–29	1.01	1.00–1.02	**0.003**	
30–39	1.02	1.01–1.02	**<0.001**	
≥ 40	0.99	0.97–1.02	0.700	
Smoking during pregnancy				0.631
No	1.01	1.00–1.02	**0.008**	
Yes	1.01	0.99–1.02	0.065	
Asthma				**0.044**
No	1.01	0.99–1.02	0.073	
Yes	1.01	1.00–1.03	**0.017**	
Covered by health insurance				0.180
No	1.04	1.01–1.08	**0.002**	
Yes	1.01	1.00–1.02	**0.003**	

Interaction *P* value adjusting for gender, age, race, PIR, maternal age at childbirth, smoking during pregnancy, asthma, health insurance status, DII, monocyte count, segmented neutrophil count, eosinophil count, and basophil count.

### 3.3. Subgroup analysis

As shown in Table 3, in the unstratified population, the constructed Crude model (OR: 1.01, 95%CI: 1.00–1.02, *P* < 0.001), Model I (OR: 1.01, 95%CI: 1.00–1.01, *P* < 0.001), and Model II (OR: 1.01, 95%CI: 1.00–1.01, *P* < 0.01) explored the association between serum PLR and the risk of ADHD, uncovering a significant positive correlation between PLR and the risk of ADHD in all three models. Furthermore, we investigated the impact of PLR on ADHD, finding that compared to the first quartile (Q1) of PLR, the second and third quartiles (Q2, Q3) considerably increased the risk of ADHD in all three models (*P* < 0.01), indicating that the risk of ADHD coincided with the increase of PLR.

**Table 3.** Associations between PLR and OR (95% CI) for ADHD medications, NHANES 2013–2014

OR (95% CI)			
Participants	Crude	Model I	Model II
All participants	1.01 (1.00–1.02) ***	1.01 (1.00–1.01) ***	1.01 (1.00–1.01) **
PLR			
Q1 (≤90.00)	Ref.	Ref.	Ref.
Q2 (90.00–116.30)	1.54 (0.59–4.03)	2.01 (0.99–4.10)	2.03 (1.02–4.02)
Q3 (>116.30)	3.7 (1.69–8.11)	4.04 (2.33–7.01)	4.17 (2.63–6.62)
P for trend	**0.001**	**<0.001**	**<0.001**

*Note*: Crude model (unadjusted). Model I adjusted gender, age, race, PIR, maternal age at childbirth, smoking during pregnancy, asthma, and health insurance status. Model II adjusted gender, age, race, PIR, maternal age at childbirth, smoking during pregnancy, asthma, health insurance status, DII, monocyte count, segmented neutrophil count, eosinophil count, and basophil count.

* *P*-value <0.05, ** *P*-value <0.01, *** *P*-value <0.001.

Subgroup analysis (Table 4) based on gender, maternal age at childbirth, and asthma demonstrated that in boys, children born to mothers aged 20–29 years at delivery, and individuals with asthma, PLR was positively linked with the risk of ADHD. This association remained significant in all models after adjusting for confounding factors (OR > 1, *P* < 0.05).

**Table 4.** Relationship between PLR and ADHD medications by gender, maternal age at childbirth, asthma (95% CI), NHANES, 2013–2014

OR (95% CI)			
Participants	Crude	Model I	Model II
Gender			
Female	0.99 (0.98–1.01)	1.00 (0.99–1.01)	1.00 (0.97–1.02)
Male	**1.01 (1.00–1.02)** **	**1.01 (1.00–1.02)** **	**1.01 (1.00–1.02)** *
Maternal age at childbirth			
< 20	1.00 (0.99–1.01)	1.00 (0.99–1.01)	1.00 (0.99–1.00)
20–29	**1.01 (1.00–1.02)** **	**1.01 (1.00–1.02)** **	**1.01 (1.00–1.02)** **
30–39	1.02 (1.01–1.02) ***	1.02 (0.99–1.04) *	1.01 (0.99–1.04)
≥ 40	0.99 (0.97–1.02)	1.57 (1.49–1.65) ***	1.08 (1.04–1.11) ***
Asthma			
No	1.01 (0.99–1.02)	1.00 (0.99–1.01)	1.00 (0.99–1.01)
Yes	**1.01 (1.00–1.03)** *	**1.02 (1.00–1.03)** **	**1.02 (1.01–1.03)** **

Note: Crude model (unadjusted). Model I adjusted gender, age, race, PIR, maternal age at childbirth, smoking during pregnancy, asthma, and health insurance status. Model II adjusted gender, age, race, PIR, maternal age at childbirth, smoking during pregnancy, asthma, health insurance status, DII, monocyte count, segmented neutrophil count, eosinophil count, and basophil count.

* *P*-value <0.05, ** *P*-value <0.01, *** *P*-value <0.001.

### 3.4. Nonlinear association between PLR and ADHD risk

**Figure 2.**
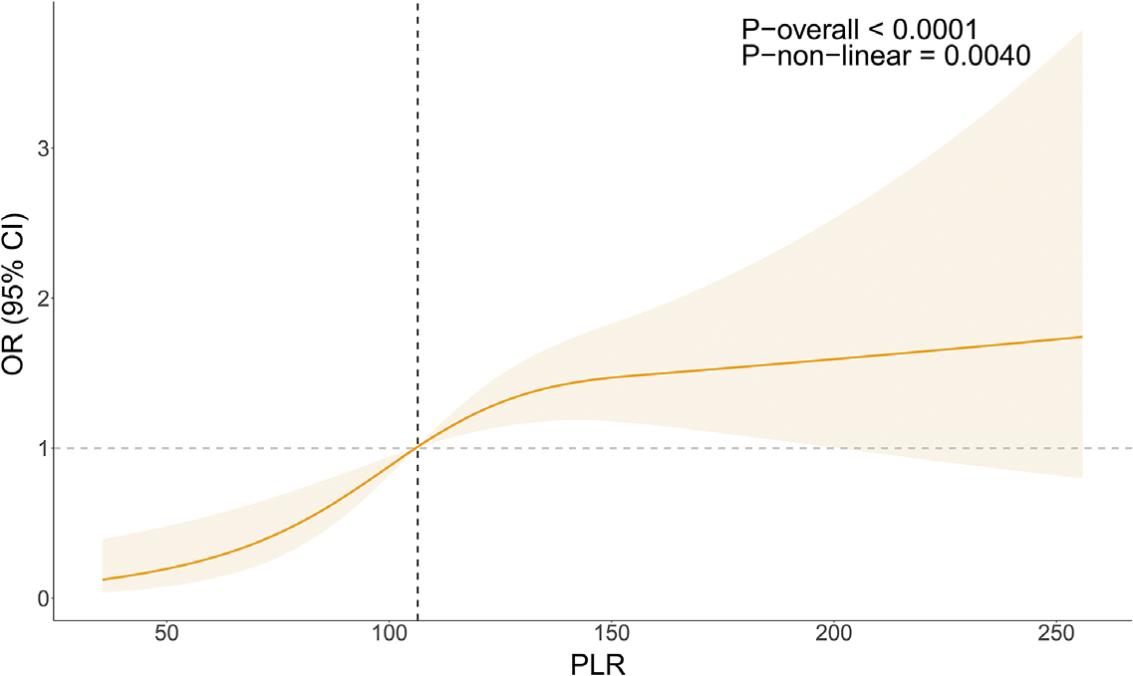
RCS graph showing the association between PLR and ADHD risk. *Note*: RCS lines are based on gender, age, race, PIR, maternal age at childbirth, smoking during pregnancy, asthma, health insurance status, DII, monocyte count, segmented neutrophil count, eosinophil count, and basophil count. OR is represented by orange lines, and the shaded area represents the 95% CI. OR: Odds Ratio; CI: Confidence Interval; PLR: Platelet to Lymphocyte Ratio; ADHD: Attention Deficit Hyperactivity Disorder.

Figure 2 illustrates the nonlinear association between PLR and ADHD risk. The RCS curve indicated a significant overall trend between PLR and ADHD risk (*P* < 0.001), and a nonlinear association was detected in models adjusting for all confounding factors (*P*-non-linear = 0.0040). As PLR increased, the risk of ADHD also increased. The RCS curve revealed that when PLR ≥ 106.40, OR > 1, a risk factor was indicated; when PLR < 106.40, OR < 1, a protective factor was indicated.

## 4. Discussion

In this project, we recruited a total of 1455 participants and recorded the measurement values of PLR in patients to explore the association between PLR and ADHD risk in the NHANES from 2013 to 2014. The results unearthed that high PLR values were linked with an increased risk of ADHD compared to the population without ADHD. Considering the potential contribution of different lifestyle factors for each participant, such as maternal smoking and drinking status during pregnancy, which may promote excessive production of inflammatory factors and affect the development of child ADHD, we also analyzed the correlation of demographic and lifestyle factors (covariate) with PLR and ADHD. Subgroup analysis demonstrated statistically significant linkages between PLR and ADHD risk in boys, children born to mothers aged 20–29 at delivery, and individuals with asthma (OR > 1, *P* < 0.05). The RCS demonstrated a nonlinear positive correlation between PLR and ADHD when PLR ≥ 106.40.

Elevated levels of pro-inflammatory cytokines in serum have been observed to interfere with the development of brain regions and neurotransmitter systems involved in executive attention, motivation, planning behavior, and decision-making.^31^ These changes in the maturation of specific brain circuits may lead to persistent neurological or neuroendocrine changes, increasing the risk of ADHD in children. Furthermore, there is evidence that inflammatory cytokines can trigger the regulation of neuroimmune mechanisms involved in behavior and emotion through the blood–brain barrier.^32^ Animal experiments have proven that allergen exposure or allergic reactions can stimulate the peripheral brain regions, as well as produce avoidance behavior, increased anxiety, and decreased social behavior.^33^ ^,^ ^34^ The symptoms of ADHD are similar to cognitive impairments caused by functional and structural changes in these two brain circuits.

Platelets are an important component of innate and adaptive immunity and are a major source of cytokines and pro-inflammatory molecules.^35^ Platelets are similar to neurons in that they participate in the uptake and release of serotonin, which is both a central neurotransmitter and a peripheral neuromodulator,^36^ thus making platelets a diagnostic tool and an interesting research model for various psychiatric disorders. Furthermore, lymphocytes play a central role in adaptive immunity, representing the regulatory or protective components of the immune system.^37^ Since PLR reflects two pathways and may be less influenced by confounding factors, it may provide more stable information than other widely used single leukocyte parameters. PLR has previously been studied in neurobehavioral disorders such as mood disorders, schizophrenia, and Alzheimer’s disease in adult patients, serving as a predictor for disease progression.^38^ ^–^^40^ Several previous studies pointed out that children with ADHD have higher PLR values compared to healthy controls,^15^ ^,^ ^16^ which is consistent with our results. In addition, our results indicated that when PLR ≥ 106.40 and OR > 1, the risk of ADHD in children was significantly increased. However, this result was not definitive, awaiting a large number of experiments for validation.

The impact of attention deficit disorders on children varies by gender, with boys being more common.^41^ This is in line with the trend observed in a previous prospective study on the relationship between maternal social psychological stress and the diagnosis of ADHD in children, where boys had a higher risk of ADHD compared to girls with their mothers under the same social stress during pregnancy.^42^ We speculated that this may be related to hormonal differences between the sexes. In the past few decades, estrogen and testosterone have been recognized to play a pivotal part in brain development as well as the occurrence and development of neurodevelopmental disorders.^43^ ^–^^45^ Estrogen is considered a key protective factor for females in dopamine-related brain disorders such as Parkinson’s disease or ADHD.^46^ ^,^ ^47^ High levels of estrogen can effectively maintain the activity of neuroprotective proteins in the brain mitochondria, reducing the incidence of neurodevelopmental disorders in females.^48^ However, after menopause, the decrease in estrogen levels considerably reduces this advantage and increases the likelihood of women developing neurodegenerative disorders such as Alzheimer’s disease.^49^ The role of androgens, however, seems to be opposite. Excessive prenatal exposure to androgens, especially testosterone, can have a tissue effect on ADHD through its impact on the dopaminergic neural circuitry, which is linked with neurodevelopmental disorders.^50^ ^,^ ^51^ A systematic analysis study on the relationship between an inflammation-related index and sex hormones revealed a positive correlation between testosterone levels and PLR,^52^ indicating that as testosterone levels increase, the PLR levels in children also increase, thereby increasing the risk of boys developing ADHD.

In this study, we uncovered that compared to other age groups, the risk of children developing ADHD seemed to be more elevated when their mothers aged 20–29 years old at delivery. People aged 20–29 are in the early stages of their careers and personal life and may face more pressure and challenges. According to a previous report from the American Psychological Association, individuals aged 20–29 in the United States have the highest levels of stress,^53^ which is tightly linked to neuroimmune. Moderate stress can activate psychological and behavioral processes. However, excessive or prolonged stressors and the resulting chronic dysregulation of the stress system can lead to a wide range of chronic pathological conditions.^54^ The impact of stress events on the immune system is an important factor in modulating the body’s ability to resist diseases.^55^ Pressure-induced stress can cause various physiological and endocrine changes, functional parameter disorders, and biochemical index changes.^56^ Persistent or recurrent stress can activate and increase the production of inflammatory cells in peripheral blood or body tissues. In addition, stress-induced changes in multiple systems in the body can also lead to disorders of various stress-related biomarkers and factors, such as abnormal elevation of inflammatory marker levels such as interleukin-1β, C-reactive protein, and tumor necrosis factor.^57^ Previous research has demonstrated that if mothers experience major stress events during pregnancy or report perceiving high levels of stress, the likelihood of children being diagnosed with ADHD increases by 1.45 to 3.03 times.^42^ Inflammation is currently considered an effective mediator between maternal psychosocial stress and adverse neurodevelopmental outcomes in offspring.^58^

Asthma is a chronic inflammatory disease with various phenotypes and endotypes. Inflammation plays a pivotal role in the development of asthma.^59^ Multiple cells and cellular components are implicated in asthma attacks. During an asthma attack, various inflammatory cells can promote the occurrence of inflammation by releasing various pro-inflammatory mediators and inflammatory molecules, thus exacerbating asthma.^60^ ^,^ ^61^ Various inflammatory biomarkers associated with asthma, such as the neutrophil-to-lymphocyte ratio, PLR, and systemic immune inflammation index, are tightly linked with the incidence and mortality of asthma patients.^62^ Previous research has unearthed that children with asthma may be more prone to experiencing mental health problems.^63^ The severity of asthma is associated with an increased risk of mental health disorders, which is more elevated in children with severe asthma.^63^ Being consistent with our findings, a large Swedish twin cohort study revealed that children with asthma have twice the risk of developing ADHD compared to those without asthma,^64^ indicating that increasing attention to children with asthma may be crucial in reducing the incidence of ADHD in the future.

The analysis of NHANES 2013–2014 data in this investigation indicated that higher PLR values were associated with an increased risk of ADHD. However, this study also has limitations. Firstly, since genetic factors have a great impact on the risk of ADHD, even if we considered race as a covariate with adjustment and further analysis, we still cannot eliminate the bias of genetic factors. Secondly, we were unable to obtain information about the mother’s lifestyle during pregnancy and their related disease reports, such as gestational diabetes and pregnancy-induced hypertension, which could potentially affect the results. Lastly, a portion of the data in this project was based on self-reported information, leading to potential subjective bias. Future research should be designed as large-scale prospective, multicenter, randomized controlled trials to confirm our findings, attempting to study intervention measures from the perspective of inflammatory mechanisms.

## 5. Conclusion

This study, based on data from the 2013–2014 NHANES, explored the association between PLR and ADHD in children aged 6–14 years. The results indicate that high PLR values are significantly associated with an increased risk of ADHD, particularly in boys, children whose mothers gave birth at ages 20–29, and those with asthma. Additionally, a non-linear positive correlation between PLR and ADHD risk was observed when PLR ≥ 106.40. These findings further support the potential role of inflammation in the onset and development of ADHD, providing preliminary evidence for PLR as a potential biomarker for ADHD and suggesting its possible use in identifying high-risk populations. However, considering the limitations of this study, future research should be designed as larger-scale, prospective, multi-center randomized controlled trials to validate these findings and further explore the relationship between inflammatory mechanisms and ADHD. Meanwhile, the clinical application potential of PLR warrants further investigation, as it may aid in the early identification of high-risk children and could inform future intervention strategies to improve the prognosis of children with ADHD.

## Data Availability

The data and materials in the current study are available from the corresponding author upon reasonable request.
